# Respiratory Muscle Performance and Pulmonary Function in Sarcopenic and Non-Sarcopenic Patients with Multiple Sclerosis: A Cross-Sectional Study

**DOI:** 10.3390/healthcare14111477

**Published:** 2026-05-27

**Authors:** Tuba Kolayli Çerezci, Filiz Eyüboğlu, Şahika Ocak, Serkan Demir, Sibel Aksu Yıldırım

**Affiliations:** 1 Department of Physiotherapy and Rehabilitation, Faculty of Health Sciences, Uskudar University, Istanbul 34662, Türkiye; filiz.eyuboglu@uskudar.edu.tr; 2 Faculty of Physical Therapy and Rehabilitation, Hacettepe University, Ankara 06100, Türkiye; sibel.aksu@hacettepe.edu.tr; 3 Department of Neurology, University of Health Sciences Sancaktepe Şehit Prof. Dr. İlhan Varank Training and Research Hospital, Istanbul 34785, Türkiye; ocaksahika07@gmail.com (Ş.O.); drsrkndemir@gmail.com (S.D.)

**Keywords:** multiple sclerosis, respiratory muscle endurance, respiratory function, sarcopenia, physiotherapy

## Abstract

**Highlights:**

**What are the main findings?**

- Secondary sarcopenia in multiple sclerosis is associated with reduced respiratory muscle endurance.
- No significant differences were observed in respiratory muscle strength parameters despite reduced respiratory muscle endurance in sarcopenic patients.

**What are the implications of the main findings?**

- Assessing respiratory muscle endurance may help detect early respiratory limitations in multiple sclerosis.
- Respiratory muscle endurance may represent a potential target for future rehabilitation studies in sarcopenic multiple sclerosis patients.

**Abstract:**

Background: Respiratory muscle dysfunction is a recognized complication of multiple sclerosis (MS) and may contribute to functional decline. Sarcopenia related to neurological impairment may further impair respiratory muscle performance in MS. This study aimed to investigate the association between sarcopenia and respiratory function, respiratory muscle strength, and respiratory muscle endurance in patients with MS. Methods: This study was designed as a cross-sectional observational study. In this study, 52 MS patients (26 sarcopenic, 26 non-sarcopenic) were evaluated. Sarcopenia was assessed using the SARC-F questionnaire, handgrip strength, bioelectrical impedance analysis, calf circumference, and walking speed (6-m walk test). Pulmonary function was evaluated using spirometry. Respiratory muscle strength was assessed using maximal inspiratory and expiratory pressures (MIP, MEP), and respiratory muscle endurance was evaluated using a constant-load test. Results: Respiratory muscle endurance (*p* < 0.001), inspiratory volume (*p* < 0.001), and forced vital capacity (*p* = 0.003) were significantly lower in sarcopenic MS patients in the primary analyses. However, inspiratory volume and respiratory muscle endurance remained significant after age adjustment, while all three parameters remained significant after EDSS adjustment. No significant differences were observed between groups in other respiratory function parameters or respiratory muscle strength including MIP, and MEP (*p* > 0.05). Conclusions: Sarcopenia is associated with reduced respiratory muscle endurance and respiratory capacity in patients with MS. These findings suggest that respiratory muscle endurance assessment may help identify sarcopenic MS patients who warrant further respiratory evaluation. Incorporating respiratory muscle endurance assessment into routine evaluation and rehabilitation strategies may be beneficial in sarcopenic MS patients.

## 1. Introduction

Multiple sclerosis (MS) is an immune-mediated disorder and results in progressive neurological impairment [1]. In addition to motor, sensory, and cognitive deficits, respiratory dysfunction may occur as a consequence of neurological involvement affecting respiratory muscles [2,3,4]. Respiratory muscle weakness and reduced pulmonary function have been reported in even in the early stages of MS and may contribute to decreased exercise capacity and functional limitations [5,6]. Respiratory muscle endurance (RME), in addition to respiratory muscle strength (RMS) and pulmonary function, has also been reported to be impaired in neurological disorders [7]. However, evidence regarding respiratory muscle endurance in MS remains limited.

Sarcopenia, traditionally associated with aging, is increasingly recognized in individuals with chronic neurological disorders, including MS. This condition is defined as secondary sarcopenia. A recently published study reveals that 1 in 5 MS patients suffer from sarcopenia [1]. Reduced physical activity, chronic inflammation, mitochondrial dysfunction, and neurological impairment may contribute to muscle mass loss and reduced muscle strength in this population [8]. Consequently, sarcopenia may represent an additional factor contributing to physical disability in patients with MS [9].

Because respiratory muscles are skeletal muscles, sarcopenia may also influence respiratory muscle performance [10]. Literature supports the idea that sarcopenia leads to decreased RMS and function [11]. Impairment of RMS and RME may negatively affect pulmonary function, and overall respiratory capacity. Although respiratory muscle dysfunction has been documented in MS, the potential contribution of secondary sarcopenia associated with neurological impairment to respiratory dysfunction in this population remains unclear [12].

Previous studies in the literature have mainly focused on global extremity and trunk muscle strength, and rehabilitation approaches have largely emphasized training of these muscle groups [13]. Respiratory dysfunction and sarcopenia have both been described in individuals with MS; however, their potential relationship has received limited attention [14,15]. In recent years, the concept of respiratory sarcopenia, which refers to the decline in respiratory muscle mass and function associated with sarcopenia, has gained increasing attention in the literature [10]. Understanding this relationship may help identify patients at higher risk of respiratory impairment and guide targeted rehabilitation strategies. Accordingly, this study aimed to investigate the association between sarcopenia and respiratory function, respiratory muscle strength, and respiratory muscle endurance in individuals with MS.

## 2. Materials and Methods

### 2.1. Ethics Approval

The study received ethical approval from the Uskudar University Non-Interventional Research Ethics Committee (61351342). Our study was conducted in accordance with the Helsinki Declaration. Written informed consent was obtained from all participants for this study.

### 2.2. Study Design and Population

This study was designed as a cross-sectional observational study and was prospectively registered on ClinicalTrials.gov (NCT07152015). No intervention or longitudinal follow-up was performed. This study was carried out at the Uskudar University Physiotherapy and Rehabilitation Application and Research Center (USFIZYOTEM) from August 2025 to February 2026. Individuals diagnosed with MS according to the 2017 McDonald criteria, aged between 18 and 60 years, without an exacerbation in the last month, and with an expanded disability status scale (EDSS) score of less than 6.5, were included in the study. In the study, two groups were formed based on the presence of sarcopenia: sarcopenic and non-sarcopenic. Patients with concomitant lung or heart disease, pregnant women, and patients with neurological diseases other than MS were excluded from this study.

**Figure 1:**
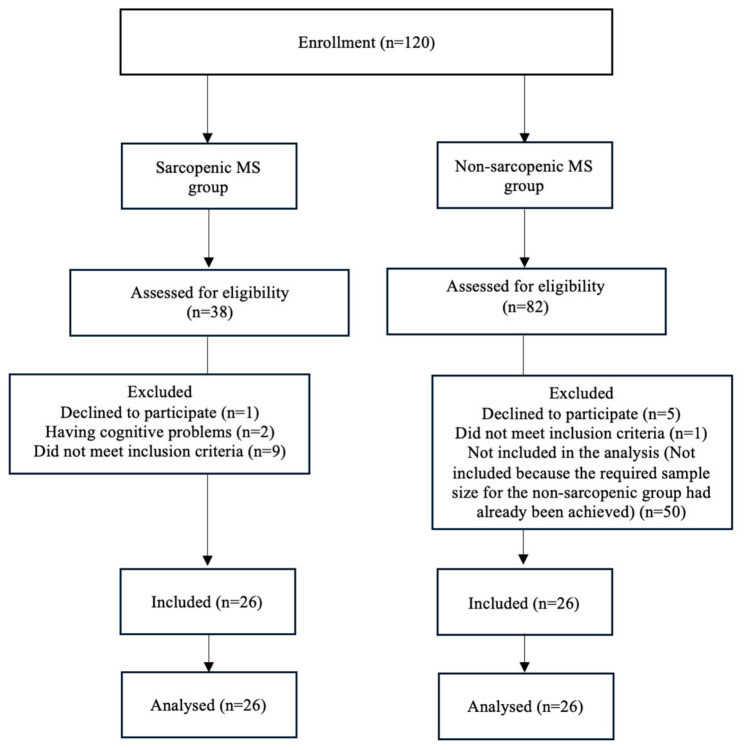
Flow diagram of the study.

Power analysis (G*Power, 3.1, Heinrich Heine University Düsseldorf, Düsseldorf, Germany) showed that, according to the MEP parameter and data reported by Sanchez-Ruiz et al., at least 46 participants were needed to achieve 95% statistical power at a 5% significance level (Cohen’s d = 1.09) [16]. RMS parameters were considered the primary outcomes of the study, whereas RME findings were interpreted as secondary exploratory outcomes. MEP was selected for the sample size calculation because expiratory respiratory muscle performance is considered to be notably affected in patients with MS. Considering a potential dropout rate of approximately 15%, the planned sample size was increased to 52 participants, with 26 individuals in each group. Participant recruitment continued until the target sample size determined by the power analysis was reached for both groups. Once the required number of non-sarcopenic participants had been achieved, additional non-sarcopenic individuals identified during the screening process were not included in the final analyses. The flow chart of the study is shown in Figure 1.

### 2.3. Measures

#### 2.3.1. Clinical Assessment and Sarcopenia Assessment

After the details of the study were explained to the participants, their demographic characteristics and MS-related information, such as EDSS score, MS type, and disease duration, were recorded.

Sarcopenia was assessed using the recommendations of the European Working Group on Sarcopenia in Older People (EWGSOP2). In this framework, sarcopenia is identified by reduced muscle strength together with decreased muscle mass or impaired muscle quality, whereas physical performance is considered an indicator of disease severity [17]. In the present study, according to EWGSOP2 recommendations, SARC-F was used as a screening tool for probable sarcopenia. Sarcopenia diagnosis was confirmed in participants with low grip strength and reduced muscle mass assessed using BIA-derived SMMI and calf circumference measurements. Gait speed was evaluated as a supportive indicator of physical performance.

The SARC-F questionnaire was initially used to self-report sarcopenia-specific symptoms, as recommended by EWGSOP2. The SARC-F is used to assess sarcopenia risk and assesses five components: muscle strength, assisted walking, stair climbing, rising from a chair, and experiencing falls. As a result of the SARC-F questionnaire, values of 4 and above are indicative of sarcopenia [18].

Handgrip strength was assessed using a JAMAR hand dynamometer (Patterson Medical, Warrenville, IL, USA) as part of the evaluation of muscle strength, one of the parameters used in sarcopenia assessment. Measurements were obtained with participants seated, with the shoulder in adduction, the elbow flexed 90°, and the forearm in a neutral position [19]. Based on the EWGSOP2 criteria, grip strength values below 27 kg for men and 16 kg for women were considered indicative of low muscle strength [17].

Muscle mass was evaluated using bioelectrical impedance analysis [20] with a body composition analyzer (Omron HBF-511B-E, Omron Healthcare Co., Ltd., Kyoto, Japan). BIA is commonly used for estimating skeletal muscle mass (SMM) in clinical and research settings [17]. Skeletal muscle mass index (SMI) was calculated as SMM divided by height squared (kg/m^2^). According to the proposed cut-off values, SMI values below 9.2 kg/m^2^ for men and 7.4 kg/m^2^ for women were considered indicative of reduced muscle mass [17,21].

Anthropometric measurements and calf circumference were recorded. Calf circumference has been reported to correlate with SMM and is commonly used as a simple indicator in the evaluation of sarcopenia. In accordance with the recommended thresholds, calf circumference values below 33 cm in women and 34 cm in men were considered indicative of reduced muscle mass [17,18].

Guidelines recommend walking speed measurements for assessing physical performance in people with sarcopenia [17,18]. Walking speed is both a safe and reliable measure of sarcopenia. This study used the 6-m walking test, commonly used in people with sarcopenia, to assess physical performance. In this test, patients were asked to walk 6 m at a normal pace, starting from a specific point. The time it took participants to walk 6 m was recorded in seconds [18]. Low physical performance was considered an indicator of severe sarcopenia, and walking speed <1.0 m/s was reported as an indicator of low physical performance for both genders [1].

#### 2.3.2. Assessment of Respiratory Parameters

Pulmonary function was assessed by a physiotherapist using a portable spirometer (Cosmed Pony FX, Rome, Italy). The parameters recorded included forced vital capacity (FVC), forced expiratory volume in one second (FEV_1_), the FEV_1_/FVC ratio, peak expiratory flow (PEF), and the forced expiratory flow between 25% and 75% of the vital capacity (FEF_25–75%_). Spirometry measurements were performed in the seated position using a nose clip in accordance with European Respiratory Society (ERS)/American Thoracic Society (ATS) recommendations. Before each testing session, device calibration was verified according to manufacturer instructions. At least three acceptable maneuvers were obtained, and the highest FVC and FEV_1_ values were recorded. Predicted spirometric values were automatically calculated by the device software using Quanjer/ERS reference equations [22].

RMS was evaluated using both static and dynamic measurements. Static RMS assessment was performed using an electronic mouth pressure device (Cosmed Pony FX, Rome, Italy), while dynamic measurements were obtained with the POWERbreathe K5 system (HaB International Ltd., Southam, UK), following the recommendations of the ERS [23,24]. For static assessment, maximal inspiratory pressure (MIP) and maximal expiratory pressure (MEP) were recorded. MIP and MEP measurements were performed according to ATS/ERS recommendations using a flanged mouthpiece and nose clip. MIP measurements were initiated from residual volume, whereas MEP measurements were initiated from total lung capacity. At least three reproducible maneuvers were performed for each parameter, and the highest value was recorded. Measurements were accepted when reproducibility criteria were achieved according to ATS/ERS recommendations. The measured values were compared with age- and sex-specific reference values and expressed as percentages of predicted values [25]. Predicted MIP and MEP values were automatically calculated by the Pony FX Quark PFT software (COSMED Pony FX, COSMED Srl, Rome, Italy) according to reference equations integrated into the system and interpreted based on published reference equations for maximal respiratory pressures [26]. Dynamic inspiratory muscle performance was evaluated using the POWERbreathe K5 device. Participants performed maximal inspiratory maneuvers from residual volume while seated and wearing a nose clip. During the maneuver, the device automatically recorded RMS index (S-Index, cmH_2_O), representing dynamic inspiratory muscle strength; peak inspiratory flow (PIF, L/s), representing maximal inspiratory airflow; and inspiratory volume (L), representing the total inspired air volume during the maneuver. At least three acceptable maneuvers were performed, and the highest values were used for analysis [27].

RME was measured with the POWERbreathe K5 (HaB International Ltd., Southam, UK) device [23]. The constant load test (CLT) was used to assess RME. According to the guidelines published by ERS, the goal of the CLT is for the individual to maintain breathing against a submaximal load until the test is completed. Participants were evaluated in a seated position while wearing a nose clip and breathing through a mouthpiece connected to the device. Before testing, participants were familiarized with the breathing procedure. The inspiratory load was set according to the testing protocol, and participants were instructed to maintain regular breathing against the applied resistance for as long as possible. The test was terminated when the participant could no longer sustain the target inspiratory load or voluntarily stopped due to fatigue. In this study, the CLT was applied at 60% of the S-Index [28]. Patients were asked to breathe in and out against a load of 60% of the S-Index and were informed that if they experienced shortness of breath, they could remove the device and terminate the test. The duration for which participants could maintain breathing against the applied load and the sustained inspiratory pressure during the test were recorded. RME was calculated as the product of inspiratory pressure and endurance time and expressed as cmH_2_O×s [29].

### 2.4. Statistical Analysis

Statistical analyses were performed using IBM SPSS Statistics software version 27.0 (IBM Corp., Armonk, NY, USA). Demographic and clinical characteristics, as well as sarcopenia assessments, were compared in MS patients with and without sarcopenia. Data distribution was assessed using the Shapiro–Wilk test and visual inspection methods. Variables with normal distribution were analyzed using Student’s *t* test, whereas non-normally distributed variables were analyzed using the Mann–Whitney U test. The T-test and Mann–Whitney U test were used for continuous variables, while the chi-square test was used to compare categorical variables. The association between RME and sarcopenia-related parameters was analyzed using Spearman’s rank correlation coefficient. Effect sizes were calculated for group comparisons. Statistical significance was set at *p* < 0.05. Additionally, multiple linear regression analysis was performed to further investigate the independent associations between sarcopenia-related parameters and RME. Additional age and EDSS-adjusted ANCOVA analyses were performed for respiratory parameters that showed significant between-group differences in the primary analyses.

## 3. Results

### 3.1. Demographic and Sarcopenia-Related Findings

At the beginning of the study, 120 MS patients were evaluated, 18 were excluded for various reasons; 6 declined to participate, 10 did not meet the inclusion criteria, and 2 had cognitive problems (Figure 1). A total of 52 sarcopenic (n = 26, 16 female, 10 male) and non-sarcopenic (n = 26, 13 female, 13 male) MS patients were included in this study. There were no significant differences between the groups in terms of sex, MS type, height, weight, BMI, smoking status, or time since last relapse (*p* > 0.05). However, sarcopenic MS patients were older (*p* = 0.006) and had a longer disease duration (*p* = 0.002) than non-sarcopenic MS patients. Additionally, significant differences were observed in sarcopenia-related parameters (*p* < 0.05, Table 1).

### 3.2. Findings Regarding Respiratory Parameters

Among the respiratory function parameters, the FVC value was significantly lower in sarcopenic MS patients in the primary analyses (*p* = 0.003). However, after adjustment for age, the between-group difference was no longer statistically significant (*p* = 0.160). No significant difference was observed between sarcopenic and non-sarcopenic MS patients in terms of FEV_1_, FEV_1_/FVC, PEF, FEF_25–75_ and predicted values (*p* > 0.05).

According to RMS results, no significant differences were observed between the groups in the absolute or predicted values of MIP and MEP (*p* > 0.05). Similarly, S-Index and PIF values did not differ significantly between the groups. However, inspiratory volume, an indicator of respiratory muscle performance, was significantly lower in sarcopenic MS patients (*p* < 0.001). RME was significantly lower in sarcopenic MS patients (*p* < 0.001). Findings regarding respiratory parameters are shown in Table 2.

### 3.3. Relationship Between Respiratory Muscle Endurance and Sarcopenia Parameters

The correlation between RME and sarcopenia-related parameters was examined using Spearman’s correlation analysis. A significant negative correlation was found between SARC-F scores and RME (r = −0.635, *p* < 0.001), indicating that higher SARC-F scores were associated with lower endurance. In addition, significant positive correlations were observed between RME and grip strength (r = 0.336, *p* = 0.015), calf circumference (r = 0.535, *p* < 0.001), and gait speed (r = 0.597, *p* < 0.001). However, no significant correlation was found between RME and SMMI (r = 0.250, *p* = 0.073) (Table 3).

Multiple linear regression analysis was performed to investigate the independent associations between sarcopenia-related parameters and RME. The model explained 22.5% of the variance in RME (R^2^ = 0.225, adjusted R^2^ = 0.176). Among the variables included in the model, only gait speed was identified as an independent predictor of RME (β = 0.430, *p* = 0.005). Grip strength and calf circumference were not significant predictors (*p* > 0.05) (Table 4).

Additional age- and EDSS-adjusted ANCOVA analyses were performed for respiratory parameters that showed significant between-group differences in the primary analyses. After adjustment for age, the between-group difference in FVC was no longer significant (*p* = 0.160), whereas inspiratory volume (*p* = 0.046) and RME (*p* < 0.001) remained significantly different between sarcopenic and non-sarcopenic MS patients. After adjustment for EDSS, the between-group differences in FVC (*p* = 0.032), inspiratory volume (*p* = 0.009), and RME (*p* < 0.001) remained significant (Table 5).

## 4. Discussion

In this study, sarcopenic MS patients showed reduced RME, whereas no significant differences were observed in RMS and predicted pulmonary function parameters between the groups. Furthermore, inspiratory volume was lower in sarcopenic MS patients. This study is the first to examine respiratory parameters in MS patients with secondary sarcopenia associated with neurological impairment. It is also one of the limited studies to contribute to the literature by evaluating RME in MS patients.

Respiratory muscle involvement has been reported in individuals with MS even at lower levels of disability. This affects respiratory capacity and respiratory efficiency [5]. Previous studies suggest that MS is associated with deterioration in respiratory function [30,31]. The interaction between MS and respiratory function remains incompletely characterized. Previous studies have reported reduced FVC values in individuals with MS, particularly in patients with higher EDSS scores and greater levels of disability [4,32]. In our study, FVC values were lower in sarcopenic MS patients in the primary analyses, whereas predicted spirometric parameters were not significantly different between the groups. However, after adjustment for age, the between-group difference was no longer statistically significant. Therefore, the observed reduction in FVC may partly reflect the potential influence of age-related and disease-related factors rather than sarcopenia alone. Previous studies have also demonstrated associations between low muscle mass and lower FVC values. Jeon et al. demonstrated that older adults at high risk of sarcopenia had significantly lower FVC values [33]. In our study, no significant difference was observed in RMS between the two groups. The lower respiratory capacity observed in sarcopenic patients may be related to factors such as postural alterations or reduced thoracic mobility, which could potentially limit effective lung expansion. However, these mechanisms were not directly evaluated in the present study.

Literature supports that sarcopenia affects RMS and leads to a decrease in both MIP and MEP values [11,33]. In our study, RMS in sarcopenic MS patients was not significantly different. RMS appeared comparable in sarcopenic and non-sarcopenic individuals with Ms. This finding may reflect the natural course of MS, which can affect respiratory muscles independently of sarcopenia. Consequently, a distinct contribution of sarcopenia to RMS could not be identified. Previous reports have also described reduced RMS in patients with MS [5,31]. Our findings also showed that inspiratory volume, which reflects the functional capacity of the muscles, was lower in the sarcopenic group. The lack of difference in MIP and MEP values between the two groups may indicate that the muscles can produce similar levels of force but lack the sustained contractile efficiency to increase inspiratory volume. Accordingly, we believe that when assessing respiratory muscle performance, functional volume measurements should be considered, not just strength parameters.

One of the most important findings of this study was the significantly reduced RME in sarcopenic MS patients. While the direct effect of sarcopenia on RME has not been demonstrated, a limited number of studies have shown that sarcopenia reduces RMS and causes a decrease in both the movement and thickness of the diaphragm muscle [10]. This suggests that sarcopenia has an impact on the respiratory system. Previous studies reporting lower RME in individuals with MS than in healthy individuals suggest that MS may affect respiratory function [4,14]. Problems such as decreased neurological stimulation to respiratory muscles such as the diaphragm, changes in the mitochondrial energy system and resulting muscle fatigue may cause a decrease in RME in MS patients [34,35]. Considering that sarcopenia causes atrophy and mitochondrial dysfunction in type I muscle fibers, fatigue in the respiratory muscles might be expected [35,36]. We think that sarcopenia may aggravate these mechanisms seen in MS and affect muscle endurance earlier than strength. However, reduced RME in MS may also be influenced by disease-related factors such as fatigue, disability level, disease duration, physical deconditioning, and neurological impairment [37]. In addition, functional measures such as SARC-F and gait speed may be affected by neurological disability independent of muscle mass loss [20]. Therefore, these factors should be considered when interpreting the observed associations.

The significant differences in respiratory parameters between sarcopenic and non-sarcopenic MS patients should be evaluated within the framework of muscle fiber type distribution and cellular adaptations. In the literature, sarcopenia is typically characterized by the selective atrophy of Type II fibers, which are responsible for high power and explosive force production [38,39]. The lower FVC and inspiratory volume values observed in the sarcopenic group in our study can be explained by the loss of these Type II fibers, particularly in the accessory respiratory and abdominal muscles that are recruited during forced expiration and maximal inspiration maneuvers. On the other hand, primary respiratory muscles such as the diaphragm predominantly consist of Type I fibers with high oxidative capacity. However, chronic inactivity and systemic inflammation associated with MS may negatively affect respiratory muscle endurance and physical performance, potentially contributing to the lower RME observed in our study [3,40]. Our current findings are consistent with the concept of “respiratory sarcopenia,” a term that has gained increasing attention in the recent literature. Respiratory sarcopenia is a clinical phenomenon characterized not only by peripheral skeletal muscle loss but also by reduced respiratory muscle mass and function, potentially leading to decreased coughing efficiency and restricted respiratory capacity [10]. Consequently, the functional decline observed in sarcopenic MS patients may represent a pathophysiological extension of the skeletal muscle atrophy process reflected in the respiratory system.

Another finding from our study was the association between RME and sarcopenia parameters such as grip strength and walking speed. RME was selected for further association analyses because it represents a functional component of respiratory performance and demonstrated significant differences between sarcopenic and non-sarcopenic patients with MS. This finding supports the impact of changes in peripheral muscle function on respiratory muscles. In addition, gait speed was identified as an independent predictor of RME in the multivariable regression analysis. Interestingly, no significant association was observed between RME and SMMI. This finding may suggest that respiratory muscle endurance in MS is influenced not only by muscle mass but also by functional performance, neuromuscular impairment, and disability-related factors. Given its effects, such as increasing exercise tolerance and delaying the onset of fatigue, RME is important for MS patients [41]. From a clinical perspective, our findings show that evaluating respiratory system function and muscle strength parameters is not sufficient in neurological diseases such as MS, and that it is also important to evaluate RME.

Several methodological considerations should be acknowledged. One limitation of this study is that some demographic and clinical characteristics, including age, disease duration, and disability level, differed between the groups. In addition, sex distribution and anthropometric characteristics showed numerical differences. Therefore, these factors should be considered when interpreting the respiratory and functional findings. Data were obtained from a single clinical center, which may limit the generalizability of the findings. In addition, the observational nature of the study design does not allow conclusions regarding causal associations between sarcopenia and respiratory parameters. A larger sample size may provide more generalizable findings.

## 5. Conclusions

In conclusion, to our knowledge, this is the first study to examine respiratory parameters in MS patients with secondary sarcopenia. Sarcopenic MS patients demonstrated lower absolute FVC values, inspiratory volume, and RME compared to non-sarcopenic patients, whereas predicted spirometric parameters were not significantly different between groups. However, no significant differences were observed in respiratory muscle strength or RMS. RME may also be associated with sarcopenia-related functional parameters in patients with MS, with gait speed identified as an independent predictor of RME. These findings highlight the potential clinical relevance of including RME assessments in the evaluation of sarcopenic MS patients and may help identify individuals who warrant further respiratory evaluation. Future intervention studies are needed to determine whether RME training improves clinical outcomes in this population.

## Figures and Tables

**Table 1:** Demographic and clinical characteristics of the participants and sarcopenia-related parameters.

	Sarcopenic MS Group (n = 26)	Non-Sarcopenic MS Group (n = 26)	
	Mean ± SD/ Median (IQR) *	Mean ± SD/ Median (IQR) *	*p* Value
Sex			0.158 ^a^
Female (n, %)	18 (69.2%)	13 (50%)	
Male (n, %)	8 (30.8%)	13 (50%)	
Age (year)	40.50 (14.75) *	33.00 (11.50) *	0.006 ^b^
Height	167.00 (10.75)	169.00 (13.00)	0.309 ^c^
Weight (kg)	71.95 (16.98) *	69.65 (10.75) *	0.876 ^b^
BMI (kg/m^2^)	26.25 (3.95) *	25.35 (3.05) *	0.667 ^b^
Smoking (pack-years)	3.5 (13.00) *	3.67 (12.38) *	0.781 ^b^
Disease duration (year)	12.50 (14.00) *	4.50 (8.18) *	0.002 ^b^
Duration since last relapse (year)	2.0 (3.75) *	2.0 (5.24) *	0.108 ^b^
EDSS, median (min–max)	2.5 (2–5)	2 (1–4)	0.006 ^b^
mean	2.94 (0.973)	2.1 (0.775)	
MS Type			0.685 ^a^
RRMS	21 (80.8%)	23 (88.5%)	
SPMS	4 (15.4%)	2 (7.7%)	
PPMS	1 (3.8%)	1 (3.8%)	
***Sarcopenia assessment***			
SARC-F	4.50 (2.0) *	0.0 (1.0) *	<0.001 ^b^
Grip strength (kg)	15.70 (5.650) *	23.10 (12.275) *	<0.001 ^b^
SMMI (kg/m^2^)	7.06 (2.528) *	7.29 (2.132) *	0.040 ^b^
Calf circumference	32.50 (1.0) *	34.75 (3.875) *	<0.001 ^b^
Gait speed (m/s)	1.114 (0.214)	1.526 (0.158)	<0.001 ^c^

* Data are presented as median (IQR); non-marked values are presented as mean ± SD. BMI: Body mass index; EDSS: Expanded Disability Status Scale; Min: minimum; Max: maximum; PPMS: Primary Progressive MS; RRMS: Relapsing-Remitting MS; SD: Standard Deviation; SMMI: Skeletal Muscle Mass Index; SPMS: Secondary Progressive MS. ^a^ Chi-square test was used, ^b^ Mann–Whitney U test was used, ^c^ Student’s *t* test was used. Normally distributed variables are presented as mean ± standard deviation, whereas non-normally distributed variables are presented as median (interquartile range).

**Table 2:** Sarcopenia-related parameter and respiratory parameter comparison between the sarcopenic MS and the nonsarcopenic MS group.

	Sarcopenic MS Group (n = 26)	Non-Sarcopenic MS Group (n = 26)			
	Mean ± SD/ Median (IQR) *	Mean ± SD/ Median (IQR) *	*p* Value	Effect Size Cohen’s d	%95 CI
***Respiratory function***					
FVC (L)	3.33 (0.782) *	3.63 (1.442) *	0.003 ^a^	0.4749	−1.015 to 0.0869
FVC (%, predicted)	91.423 (10.416)	94.115 (8.214)	0.306 ^b^	−0.2870	−0.84 to 0.27
FEV_1_ (L)	2.725 (0.594)	3.077 (0.712)	0.059 ^b^	−0.5369	−1.065 to 0.0407
FEV_1_ (%, predicted)	89 (14.750) *	90.50 (11.750) *	0.755 ^a^	0.0518	−0.49 to 0.59
FEV_1_/FVC	81.538 (8.496) *	78.885 (10.798) *	0.515 ^a^	−0.1065	−0.65 to 0.44
FEV_1_/FVC (%, predicted)	80.50 (17.250) *	82 (11.50) *	0.304 ^a^	−0.1672	−0.71 to 0.38
PEF (L/s)	4.764 (1.494)	5.315 (1.857)	0.244 ^b^	−0.3268	−0.87 to 0.23
PEF (%, predicted)	63.846 (15.813)	63.654 (17.550)	0.967 ^b^	0.0115	−0.53 to 0.56
FEF_25–75%_ (L/s)	2.971 (1.045)	3.391 (1.071)	0.158 ^b^	−0.3973	−0.94 to 0.15
FEF_25–75%_ (%, predicted)	76.923 (22.067)	77.577 (20.078)	0.911 ^b^	−0.0310	−0.57 to 0.51
***Respiratory muscle strength assessment***					
MEP (cmH_2_O)	70.808 (20.058)	83.346 (27.188)	0.064 ^b^	−0.5248	−1.047 to 0.057
MEP (%, predicted)	71.115 (20.202)	70.654 (16.807)	0.929 ^b^	−0.0248	−0.57 to 0.52
MIP (cmH_2_O)	84.077 (25.221)	93.346 (23.384)	0.175 ^b^	−0.3811	−0.93 to 0.17
MIP (%, predicted)	109.615 (31.925)	110.846 (37.726)	0.899 ^b^	−0.0352	−0.58 to 0.51
S-Index (cmH_2_O)	52 (11) *	56.50 (17.750) *	0.087 ^a^	0.2781	−0.82 to 0.27
PIF (L/s)	3 (0.700) *	3.30 (1.00) *	0.067 ^a^	0.2973	−0.84 to 0.25
Inspiratory volume (L)	2 (0.950) *	2.30 (0.475) *	<0.001 ^a^	0.5370	−1.07 to 0.003
***Endurance assessment***					
Respiratory muscle endurance (cmH_2_Oxsn)	2070.115 (848.352)	5623.523 (2697.560)	<0.001 ^b^	−1.7771	−2.411 to −1.121

* Data are presented as median (IQR); non-marked values are presented as mean ± SD. SD: standard deviation; FEV_1_: forced expiratory volume in one second; FVC: forced vital capacity; PEF: peak expiratory flow; PIF: peak inspiratory flow; FEF_25–75%_: forced expiratory flow from 25% to 75%; MIP: maximal inspiratory pressure; MEP: maximal expiratory pressure. ^a^ Mann–Whitney U test was used, ^b^ Student’s *t* test was used. Effect sizes are presented as Cohen’s d with 95% confidence intervals for parametric comparisons and as rank biserial correlation coefficients for non-parametric comparisons. Normally distributed variables are presented as mean ± standard deviation, whereas non-normally distributed variables are presented as median (interquartile range).

**Table 3:** Correlations between respiratory muscle endurance and sarcopenia parameters in MS patients.

	r	*p* Value
SARC-F	–0.635	<0.001
Grip strength (kg)	0.336	0.015
SMMI (kg/m^2^)	0.250	0.073
Calf circumference	0.535	<0.001
Gait speed (m/s)	0.597	<0.001

r: Spearman rho correlation coefficient; SMMI: skeletal muscle mass index.

**Table 4:** Multiple linear regression analysis of sarcopenia-related parameters associated with respiratory muscle endurance in patients with multiple sclerosis.

Predictor	B	SE	Standardized β	t	*p*
Grip strength (kg)	19.7	60.6	0.054	0.325	0.746
Calf circumference	33.2	138.7	0.037	0.239	0.812
Gait speed (m/s)	4334.3	1476.2	0.430	2.936	0.005 *

Model statistics: R^2^ = 0.225; Adjusted R^2^ = 0.176. Abbreviations: B, unstandardized coefficient; SE, standard error; β, standardized beta coefficient. * *p* < 0.05.

**Table 5:** Age and EDSS-adjusted ANCOVA analyses for respiratory parameters showing significant between-group differences.

	Age-Adjusted				EDSS-Adjusted			
	Group F	Group p	Age F	Age p	Group F	Group p	EDSS F	EDSS p
FVC	2.04	0.160	7.50	0.009	4.85	0.032	0.116	0.735
Inspiratory volume	4.20	0.046	0.918	0.343	7.33	0.009	0.593	0.445
RME	39.36	<0.001	1.71	0.197	39.22	<0.001	1.54	0.220

ANCOVA: Analysis of covariance; FVC: Forced vital capacity; RME: Respiratory muscle endurance. Statistical significance was set at *p* < 0.05.

## Data Availability

The datasets generated and/or analyzed during the current study are available from the corresponding author on reasonable request. The dataset includes sensitive health-related information derived from MS patients. In accordance with data protection regulations (including the Turkish Personal Data Protection Law, KVKK) and ethical considerations, the data are not publicly available but can be shared under controlled conditions upon reasonable request. Participants provided informed consent indicating that their data would be used solely for research purposes and could be shared with appropriate permissions under controlled conditions.
